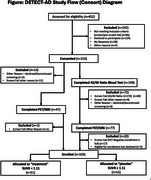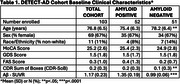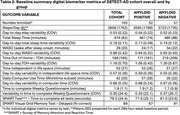# DETECT‐AD (Digital Evaluations and Technologies Enabling Clinical Translation for Alzheimer’s Disease): Baseline results of a simulated anti‐amyloid clinical trial using digital biomarkers

**DOI:** 10.1002/alz70859_106111

**Published:** 2025-12-25

**Authors:** Jeffrey A Kaye, Alex B Speers, Amanda B Mar, Jennifer Marcoe, Nora Mattek, Wan‐Tai Michael Au‐Yeung, Zachary T Beattie, Sarah Gothard, Elise J Hanna, Aimee Pierce, Lisa C Silbert, Yanan Shang, Daniel Schwartz, Joel S. Steele, Chelsea A Thomas, Kirsten M Wright

**Affiliations:** ^1^ Oregon Health & Science University, Portland, OR USA; ^2^ NIA‐Layton Aging & Alzheimer's Disease Center, Portland, OR USA; ^3^ Oregon Center for Aging & Technology (ORCATECH), Portland, OR USA; ^4^ Portland Veterans Affairs Medical Center, Portland, OR USA; ^5^ University of North Dakota, Grand Forks, ND USA

## Abstract

**Background:**

Current conventional cognitive testing and self‐report questionnaires used in clinical trials for Alzheimer’s disease (AD) especially in prodromal early stages are poorly sensitive to detect subtle and gradual clinical change as well as lack ecological validity. Digital biomarkers (DBs) can detect salient change objectively, continuously and unobtrusively in real life settings, thus dramatically improving the conduct of clinical trials. Despite this promise there are few trial‐focused studies conducted in the community that have provided evidence for how well continuously assessed, home‐based digital biomarkers may detect clinically meaningful changes in individuals with changing amyloid (Aβ) burdens in pre‐symptomatic and prodromal AD. DETECT‐AD (ClinicalTrials.gov, NCT05385913) is a simulated clinical trial being conducted to create this evidence. Here we report the baseline results of the study.

**Method:**

Clinically pre‐symptomatic and prodromal AD patients with estimable rates of AD progression based on Aβ Florbetapir‐PET status are enrolled and divided into n=50 Aβ “positive” (higher amyloid burden) patients who predictably progress (as if they were receiving placebo) and n=50 Aβ “negative” patients progressing more slowly (representing the treatment group). A study‐provided multivitamin mimics trial conditions and study drug adherence behavior. Baseline standard clinical and biomarker measures are obtained along with DBs captured with passive and active sensors (actigraphy, bed mats, IR sensing, e‐pillbox, computing device use, weekly online reporting/cognitive assessment). Primary outcome is the change in a composite DB composed of measures in 4 domains: mobility, cognition, sleep, and socialization. Secondary measures are the relationship of DBs to fluid biomarkers (plasma ptau181/217, amyloid, NfL), MRI, and PET measures.

**Result:**

Full enrollment (n=103) was achieved (see Figure). Baseline clinical characteristics are presented in Table 1. Primary baseline DBs (average of 4 four weeks) are presented in Table 2. By design, differences were noted at baseline in SUVR. At baseline there were differences in age (2.8 years) and CDR SoB (0.1 points) in clinical characteristics. There were no differences in DBs during the baseline period.

**Conclusion:**

Digital biomarkers can be integrated into anti‐amyloid designed trials. Stable baseline DB measures between Aβ “negative” and “positive” patients sets the stage for sensitively observing divergent DB trajectories over time.